# Visuospatial cognitive tests for the evaluation of patients with Parkinson's disease

**DOI:** 10.1590/S1980-57642009DN20300007

**Published:** 2008

**Authors:** Marina Ceres Silva Pena, Emmanuelle Silva Tavares Sobreira, Carolina Pinto Souza, Guiomar Nascimento Oliveira, Vitor Tumas, Francisco de Assis Carvalho do Vale

**Affiliations:** 1 Psicóloga, Pesquisadora do Grupo de Neurologia Comportamental (GNC) do Hospital das Clínicas da Faculdade de Medicina de Ribeirão Preto da Universidade de São Paulo (HCFMRP-USP), Mestranda do Programa de Pós-Graduação em Saúde Mental da Faculdade de Medicina de Ribeirão Preto, Universidade de São Paulo (PGSM-FMRP-USP).; 2 Neurologista do Hospital das Clínicas da Faculdade de Medicina de Ribeirão Preto da Universidade de São Paulo (HCFMRP-USP).; 3 Neurologista, Professor Doutor da Faculdade de Medicina de Ribeirão Preto da Universidade de São Paulo (FMRP-USP), Coordenador do Ambulatório de Distúrbios do Movimento (AEXP) do Hospital das Clínicas da Faculdade de Medicina de Ribeirão Preto da Universidade de São Paulo (HCFMRP-USP).; 4 Neurologista, Coordenador do Grupo de Neurologia Comportamental (GNC) do Hospital das Clínicas da Faculdade de Medicina de Ribeirão Preto da Universidade de São Paulo (HCFMRP-USP).

**Keywords:** Parkinson's disease, visuospatial functions, cognitive tests

## Abstract

Parkinson's disease (PD) is a neurological disorder characterized by motor disturbances, neuropsychological symptoms and cognitive changes, including cases of dementia. The most frequently described cognitive changes in these patients involve executive and visuospatial functions, which are very important for the execution of daily life activities.

**Objective:**

To compare different tests used to examine visuospatial functions in patients with PD.

**Methods:**

Thirty-five patients (21 women) with PD symptoms (medicated and “on”) and mean schooling of 5.5±4.2 years were examined using the following tests: *Mini-Mental State Examination* (MMSE), *Dementia Rating Scale* (DRS), *Scales of Outcomes of Parkinson's Disease* (SCOPA-COG), *Hooper Visual Organization Test* (HVOT), *Judgment of Line Orientation, Form V* (JLO), and *Clock drawing task – CLOX* (1 and 2).

**Results:**

The mean MMSE score was 24.8±3.03and 54.8% of the patients performed correctly in the copy of a pentagon drawing, with a medium-level performance in most tests. Good correlations were detected between JLO versus SCOPA *Assembling patterns* (0.67), JLO versus HVOT (0.56), JLO versus CLOX2 (0.64), SCOPA *Figure Composition* versus HVOT (0.54), CLOX1 versus CLOX2 (0.43), and DRS *Construction* versus CLOX2 (0.42).

**Discussion:**

Although correlations were detected, not all were strong, probably because the tests employed do not measure solely visuospatial functions, but also other skills such as attention, motor ability and executive functions. A limitation of the present study was the lack of a control group for the establishment of adequate standards for this population.

## Abstract (pt)

A doença de Parkinson (PD) é um transtorno neurológico caracterizado por distúrbios motores, sintomas neuropsicológicos e por alterações cognitivas, inclusive casos de demência. As alterações cognitivas mais descritas nesses pacientes são as funções executivas e as visuais-espaciais, funções importantíssimas para a realização de atividades da vida diária.

**Objetivo:**

Comparar diferentes testes que examinam funções visuais-espaciais em pacientes com DP.

**Métodos:**

Foram avaliados 35 pacientes (21 mulheres) sintomáticos para DP (medicados e em “on”) e escolaridade média de 5,5±4,2 com os seguintes testes: *Mini-Exame do Estado Mental* (MEEM), Escala de Avaliação de Demência de Mattis (EADM), Escala de Conseqüências da Doença de Parkinson (SCOPA-COG), Teste de Organização Visual de Hooper (TOVH), Julgamento de Orientação de Linhas – Forma V (JOL), e Teste do Desenho do Relógio – CLOX (1 e 2).

**Resultados:**

Os pacientes obtiveram média 24,8±3,03 no MMSE e 54,8% acertou o de cópia do desenho do pentágono e desempenho mediano na maioria dos testes. Foram encontradas boas correlações entre JOL versus SCOPA *Composição de Figuras* (0.67), JLO versus TOVH (0.56), JOL versus CLOX2 (0.64), SCOPA *Composição de Figuras* versus TOVH (0.54), CLOX1 versus CLOX2 (0.43) e EADM *Construção* versus CLOX2 (0.42).

**Discussão:**

Mesmo tendo sido verificadas correlações, nem todas foram “fortes”, o que pode ser explicado pelo fato de que os testes não medem somente funções visuais-espaciais, mas outras habilidades como atenção, habilidade motora e funções executivas. Uma dificuldade do presente estudo diz respeito à falta de grupo controle para estabelecer padrões adequados à essa população.

Parkinson's disease (PD) is the second most common neurodegenerative disease, affecting around 3% of Brazilians older than 64 years of age.^1^ The disease is mainly characterized by the classic motor symptoms: resting tremor, rigidity, bradykinesia and postural instability. Nevertheless, PD is also associated with many other non-motor manifestations that predominate at the late stage of the disease and significantly impair the quality of life of the affected individuals.

Behavioral and cognitive abnormalities are very frequent clinical problems in PD. Around eighty percent of PD patients develop cognitive abnormalities that are detectable during the course of the disease.^2-5^ Some 83% of PD patients that survive to the 20^th^ year of the disease develop dementia.^6^

Executive functions and visuospatial abilities are frequently impaired in patients with PD.^2,4^ These executive functions are characterized by principles of cognitive organization and mental processes involved in the changing situations of daily life. They are adjustable skills that modify mental strategies in response to environmental contingencies in order to reach an efficient and adequate solution for these situations.^7^ Executive functions are skills that require planning and execution of activities including task initiation, attention, concentration, selectivity of stimuli, abstraction ability, planning, flexibility, mental control, self-control, working memory, and impulse inhibition.^7^

Visuospatial functions are active perceptual skills modulated by attention, which in turn is also modulated by perception. Attention also involves reflection at different levels, such as selectivity, division and sustained attention.^8^ Visuospatial functions are skills needed to understand visual representations and their spatial relations; ability incorporated in the execution of daily life activities; ability to move in our environment with appropriate orientation, as well as the ability to find objects in our visual field or in different points in space.

It is very important to examine these cognitive functions in patients with PD.

The aim of the present study was to evaluate the performance of patients with PD on cognitive tests that explore visuospatial functions, and to examine the correlations between complex and more rudimentary bedside cognitive tests.

## Methods

### Patients and procedures

We evaluated consecutive patients with PD that attended an outpatient Movement Disorders Clinic at the Ribeirão Preto School of Medicine Hospital. Inclusion criteria were diagnosis of PD^9^ and possibility to return at a later date to complete the cognitive evaluation. Patients without schooling were included if they were not illiterate and could read and write. Exclusion criteria were: presence of psychotic symptoms, score on the 15-item Geriatric Depression Scale suggestive of depression^10^ and the use of antidepressive, anxiolytic and/or anticonvulsant drugs.

The clinical assessment of the patients was conducted in two stages. First, the patient was evaluated by a neurologist, who applied the Unified PD Rating Scale (UPDRS), Hoehn & Yahr and Schwab & England scales, Mini Mental State Examination, Survey of Subjective Memory Complaints, Frontal Assessment Battery, Verbal Fluency, Digit Span (DO and IO), Word List, Clock Drawing Task (CLOX 1 and 2), Interlocking Finger Test, Pfeffer Questionnaire and Clinical Dementia Rating. The patients were reevaluated at a later date, with a maximum interval of 30 days from the first evaluation. Patients were assessed by a psychologist who applied the Wisconsin Card Sorting Test, Dementia Rating Scale, Hooper Visual Organization Test, Judgment of Line Orientation-Form V, Scale of Outcomes of Parkinson Disease-Cognitive and the Neuropsychiatry Inventory. The first evaluations were performed in around 45-60 minutes, while the second evaluations were performed in 90–120 minutes.

For clinical evaluation we used a shortened version of the UPDRS motor subscale containing only 8 items. This shortened version scored the same signs evaluated by the Short Parkinson's Evaluation Scale^11^ but used the original 5-point items of the UPDRS. This scale was proven to have good reliability and validity in Brazilian patients with PD.^12^ This study was approved by the local ethics committee and patients included in this study gave their informed consent to participate.

### Visuospatial function – Cognitive tests

#### Mini-Mental State Examination (MMSE)

This is a brief tool used for screening of cognitive impairment. The copy of the *pentagon drawing* item was used as a test of visuospatial function.^13^

#### Mattis Dementia Rating Scale (MDRS)

This scale is used to assess cognitive status in general, widely employed as an aid for the diagnosis of dementia. It assesses attention, initiation and perseveration, construction, conceptualization, and memory. Its objective is to detect losses in the memory, language, orientation, attention, and praxis domains. The test has been standardized and validated in Brazil. For the present study we used the *Construction* subscale (*MDRS-constr*). In this subscale the patient is asked to copy geometric shapes and write their name.^14^

#### Scale of Outcomes of Parkinson Disease (SCOPA-COG)

This scale assesses cognitive function in patients with PD, measuring the following domains: memory and learn ing, attention, executive functions, visuospatial functions, and memory. For the present study, we analyzed only the *Figure Composition* subscale. In this subscale, the patient is shown 5 incomplete patterns and has to choose 2 or 3 shapes out of 4 or 5 possible alternatives to complete the pattern.^15^

#### Hooper Visual Organization Test (HVOT)

This test assesses the ability to rearrange conceptual figures (30) that have been defragmented and disarranged. It assesses perceptual differentiation and conceptual reorganization (including mental rotation) of object fragmentation.^16^

#### Judgment of Line Orientation, Form V (JLO)

This test examines the ability to estimate angular relations between line segments (30 pairs) by pairing them visually with numbered lines grouped in a semicircle. It assesses spatial orientation.^17^

#### Clock Drawing Task – CLOX (1 and 2)

The objective of the clock drawing task is to examine planning, constructive praxis, and visuospatial attention. The first part of the test (CLOX1) measures performance of the patient in drawing a clock with numbers and hands set to “1:45”. In the second part of the test (CLOX2), the examiner draws the numbers 12, 6, 3 and 9 on a pre-existing circle, sets the hands again to “1:45” and the patient is allowed to copy the examiner's clock.^18,19^

### Statistical analysis

The variables were processed using a database and statistical analysis carried out using the SPSS 13.0 software. Firstly, all variables were analyzed from a descriptive viewpoint, with calculation of means, standard deviations, and minimum/maximum values of the quantitative data. For categorical variables, relative and absolute frequencies were calculated. Non-parametric testing was used because variables did not present a normal distribution. Spearman's correlation coefficient was used to study the correlations between cognitive tests, where the level of significance used for the test were 5% and 1% (p<0.05, 95% or p<0.01, 99% confidence interval).

## Results

The clinical, demographic characteristics and results of the cognitive tests of the patients evaluated are presented in Table 1. The correlations between the cognitive tests are presented in Table 2.

**Table 1:** Clinical and demographic characteristics, and results of cognitive tests in PD patients.

Patients evaluated	45
Gender (men:women)	24:21
Age (mean±SD)	62.3±12.3 years (range: 34 to 87 years)
Age at PD presentation	55.22±12.9 years (range: 28-83 years)
Duration of disease	6.8±4.2 years (range: 2 to 17 years)
Schooling (years) (mean±SD)	5.62±12.9 years (range: 0-19 years)
Hoehn and Yahr	2±0.7 (range: 0-3)
Schwab and England	79%±17 (range: 50-100)
Motor UPDRS (shortened version)	12.2±6.8 (range: 4 to 28)
*MMSE*	24.6±3.9 (range: 9-29)
*MMSE pentagon drawing*	46.7% were not able to draw
*MDRSl*	122.6±17.7 (range: 37-141)
*MDRS-constr subscale*	3.93±2.02 (range: 0-6)
*SCOPA-COG total score*	18.75±7.0 (range: 0-30)
*SCOPA-COG figcomp subscale *	2.64±1.58 (range: 0-5)
*HVOT*	11.81±5.2 (range: 3-21)
*JLO*	18.98±7.09(range: 0-28.5)
*CLOX 1*	7.9±3.2 (range: 0-14)
*CLOX 2*	10.9±2.8 (range: 2-15)

HVOT, Hooper Visual Organization Test; JLO, Judgment of Line Orientation, Form V; MMSE, Mini-Mental State Examination; MDRS, Mattis Dementia Rating Scale; MDRS constr, Mattis Dementia Rating Scale construction subscale; SCOPA-COG, Scale of Outcomes of Parkinson Disease-Cognitive; SCOPA-COG figcomp, Scale of Outcomes of Parkinson Disease-Cognitive Figure Composition subscale; CLOX 1, Clock drawing task part 1; CLOX 2, Clock drawing task part 2.

**Table 2:** Correlations between cognitive tests (Spearman correlation coefficient (Rho)).

	MDRS Constr	MDRS Total	HVOT	JLO	SCOPAcog figcomp	SCOPAcog Total	MMSE pentagon	MMSE Total	CLOX1	CLOX2
MDRS Constr		0.482** 0.001			0.345* 0.022	0.304* 0.045		0.339* 0.033		0.383* 0.018
MDRS TOTAL	0.482** 0.001		0.616** 0.000	0.452** 0.002	0.532** 0.000	0.762** 0.000	0.362* 0.025	0.636** 0.000	0.504** 0.001	0.537** 0.001
HVOT		0.616** 0.000		0.537** 0.000	0.436** 0.003	0.568** 0.000			0.427** 0.008	0.486** 0.002
JLO		0.452** 0.002	0.537** 0.000		0.620** 0.000	0.476** 0.001				0.594** 0.000
SCOPAcog figcomp	0.345* 0.022	0.532** 0.000	0.436** 0.003	0.620** 0.000		0.689** 0.000			0.439** 0.007	0.552** 0.000
SCOPAcog TOTAL	0.304* 0.045	0.762** 0.000	0.568** 0.000	0.476** 0.001	0.689** 0.000		0.331* 0.046	0.547** 0.000	0.436** 0.007	0.398* 0.015
MMSE pentagon		0.362* 0.025				0.331* 0.046		0.529** 0.001		
MMSE	0.339* 0.033	0.636** 0.000				0.547** 0.000	0.529** 0.001			
CLOX1		0.504** 0.001	0.427** 0.008		0.439** 0.007	0.436** 0.007				0.555** 0.000
CLOX2	0.383* 0.018	0.537** 0.001	0.486** 0.002	0.594** 0.000	0.552** 0.000	0.398* 0.015			0.555** 0.000	

* Correlations significant at 0.05 level;

** Correlations significant at 0.01 level.

We found no correlation between duration of the disease, the shortened score of motor UPDRS, Hoehn and Yahr stage, Schwab and England functional capacity and cognitive tests.

The total scores on the MMSE, SCOPA-COG and MDRS were highly correlated. Of these composite scales, the MDRS was the scale which correlated with most specific visuospatial tests, followed by the SCOPA-COG and finally the MMSE.

The *SCOPA-COG Figure Composition* subscale was the test that presented most significant correlations with the other visuospatial tests, followed by the JLO and HVOT.

Among the more basic bedside tests, the CLOX^2^ presented the most significant correlation with the most complex visuospatial tests.

## Discussion

The patients in this sample obtained low mean scores in all tests applied to assess visuospatial functions. If we consider the inability to copy the pentagon drawing as evidence of visuospatial dysfunction, we could classify 62.2% of the patients as presenting abnormalities in this cognitive field. We should not rule out that this could be explained by the low schooling of the individuals examined since schooling is known to influence performance in tests such as those applied here.^14,20^

A limitation of the present study was the lack of a control group to be used to establish adequate standards for the population studied, as well as the lack of a statement declaring the test as the “gold standard” for the assessment of this function. Another important point is the lack of adaptation, validation and standardization of the tests applied, hampering comparison with other studies, and the need for a detailed analysis of the results obtained, indicating the need for further studies in this area.

We found no correlation between the performance in the cognitive tests and patient age, duration of the disease, severity of motor symptoms, stage of the disease and functional capacity. This was finding was unexpected and could be explained by the small number of patients evaluated in this sample.

Several correlations were detected between the tests evaluated, most of which were moderate. Even though correlations were detected, not all were strong, possibly due to the fact that these tests do not measure only visuospatial functions, but also other skills such as attention, motor ability, and executive functions. This indicates the important relationship between visuospatial and executive functions, as well as the need to apply tasks that do not depend on motor function when testing visual perception in patients with combined extrapyramidal and cognitive impairment, as is the case for patients with PD.^21^

The scales for global assessment were all correlated, but the MDRS had more correlations with specific tests of visuospatial functions, suggesting that this scale is better than the SCOPA-COG and the MMSE for measuring this cognitive function in patients with PD. However, the *SCOPA-COG Figcomp* subscale was also highly correlated with the specific scales, suggesting that it may be an option for evaluating visuospatial function in patients with PD

The CLOX 2 was the simplest test to correlate significantly with more complex tests of visuospatial function. The CLOX test is easy to administer, has an internally consistent measure, and good reliability, and has been strongly associated with MMSE scores, in addition to being useful for discriminating between patients with and without Alzheimer's disease.^19,21^

Lastly, there were significant correlations with MDRS, a scale widely used to assess cases of dementia, and with SCOPA-COG, a practical and short scale specifically developed to assess the cognitive deficits typical of PD.^15^

The visuospatial deficits of patients with PD are a hotly debated issue due to the discrepancy of data obtained from different studies investigating them. This disparity may be partially attributed to the pooling of different cognitive tasks and mechanisms as measures of the same visuospatial functions.^21^
